# Supplementary material for: SLCO1B1 Exome Sequencing and Statin Treatment Response in 64,000 UK Biobank Patients
Source: Int J Mol Sci. 2024 Apr 17;25(8):4426. doi: 10.3390/ijms25084426 (PMC11050003; doi:10.3390/ijms25084426)
Supplement: Supplementary file 1 [file ijms-25-04426-s001.zip › File S1.supplementary_information.SLCO1B1_exomes_2024.04.17.pdf]

SLCO1B1 exome sequencing and statin treatment response in 64,000 UK Biobank patients

Deniz Türkmen et al.

Supplementary information

Table of Contents

SLCO1B1 exome sequencing and statin treatment response in 64,000 UK Biobank patients 1
METHODS 2
Outcomes 2
Baseline 2
Statistical Analysis 2
General Practice (GP) Data for common variants 2
TWIST 2
RESULTS 3
Comparison between baseline self-report and GP-prescribed statins 3
Common *SLCO1B1* variants 3
Rare variants in *SLCO1B1* identified by exome sequencing 3
REFERENCES 5

METHODS

Outcomes

Baseline

High LDL is >2.86 mmol/L(1).

Statistical Analysis

General Practice (GP) Data for common variants

Participants entered the model after the date of first prescription, exited on the date of first incident outcome or end of records for CHD and stroke. This 'intention-to-treat' approach minimized the impact of genetically influenced discontinuation on cardiovascular outcomes, allowing us to assess those events even if the patient has discontinued due to an adverse event.

The discontinuation model considered the period from the first prescription to the discontinuation date, if available. *SLCO1B1**5 had been found to increase discontinuation risks in women but not in men previously (2), we stratified by sex for the discontinuation models. For muscle symptom analysis, patients were censored at the first incident outcome. We also separately analyzed muscle symptoms in patients who were on statins for at least five years, considering previous research (3) linking longer statin use to increased muscle symptoms. This extended duration allowed sufficient time for such events to occur.

TWIST

The method includes several ‘Genetically Moderated Treatment Effect’ (GMTE) analyses:. In this study we considered `GMTE0’ (the genetic effect in untreated people); GMTE1 (the genetic effect in treated people); RGMTE (the GMTE1 effect minus the GMTE0 effect); MR (which uses the gene as an instrumental variable) and RGMTE-MR (a weighted average of the MR and RGMTE estimates). From these analyses, the most efficient and robust estimate of the GMTE is derived, which in this case were the RGTME and RGMTE-MR for this study. The TWIST framework explicitly tests the association between genotype and outcome in the treated and untreated groups separately, to determine the GMTE independent of any effect in untreated individuals. We used R version 4.2.1 and R package {twistR} (https://github.com/lukepilling/twistR) v.0.1.4.

RESULTS

Comparison between baseline self-report and GP-prescribed statins

The flowchart in Figure 1 in the manuscript shows how we arrive at our two analysis samples: 67,630 participants self-reported simvastatin or atorvastatin use at the baseline, and 65,513 participants were prescribed simvastatin or atorvastatin in the available primary care data. Though these numbers seem quite similar, the overlap in participants included in both analyses is only 29,545 due to the differences in sample overlap and timeframes (see Methods section of details of ascertainment of the two groups).

We identified 35,435 UK Biobank participants who did not self-report statin use at the baseline assessment with a statin prescription in the primary care record, where the date of first prescription is later than the baseline assessment date. This explains the apparent disconnect between baseline and GP prescriptions: the majority of prescriptions occur after assessment (this is to be expected, as the participants increase in age, so does cardiovascular preventive medicine use). We also identify 4,483 UK Biobank participants who did not self-report statin use at the baseline assessment but have a statin prescription prior to their baseline assessment date. This suggests failure to self-report, but the overall % is very low.

Common *SLCO1B1* variants

Total cholesterol and triglycerides levels were higher for *SLCO1B1**5 and *15 carriers versus non-carriers (Cholesterol: $Coef_{*15}$:0.05, 95%CI: 0.03 to 0.07, $p=10^{-6}$ and $Coef_{*5}$:0.1, 95% CI: 0.06 to 0.15, $p=3x10^{-6}$, and triglycerides: $Coef_{*15}$:0.05, 95% CI: 0.03 to 0.07, $p=2.5x10^{-5}$ and $Coef_{*5}$: 0.12, 95%CI: 0.07 to 0.18, $p=2x10^{-5}$).

Rare variants in *SLCO1B1* identified by exome sequencing

In combined analysis, 24 participants carried at least one allele of the four LDL increasing rare variants: 45.8% were female with a mean age of 61.64 (SD 3.5) at the baseline. Those 24 participants had higher c-LDL (0.76 mmol/L, 95%CI 0.48 to 1.03, $p=7x10^{-8}$) compared to non-carriers (those without these 4 rare variants). Of the 24 carriers, 45.8% had clinically high LDL (>3.5mmol/L) compared to 11.2% in non-carriers (Supplementary Table11). While in men the rare variants increased the c-LDL compared to non-carriers (Coef 1.77, 95% CI 1.21 to 2.33, $p= 5.6x10^{-10}$), they did not in women (Coef 0.39, 95% CI -0.23 to 1, p=0.2).62.5% of patients carrying the variants reported experiencing pain at the baseline assessment; of those participants, the majority were men (66.6%). Male participants carrying the LDL increasing

rare variants had increased risk for experiencing pain while on statins compared to non-carriers (OR=1.39, 95% CI=1.06 to 1.82, *p*=0.002). It was not significant for women (OR=1.02, 95% CI: 0.8 to 1.36, *p*=0.91).

In the remaining 43 variants not associated with LDL, some were associated with other relevant biomarkers (Supplementary Table S9): direct bilirubin levels were increased with stop gained variant 12:21222355:C:T (mac=70, Coef: 0.53, 95% CI: 0.3 to 0.76, $p=9.3x10^{-6}$), splice donor variant 12:21224840:G:A (mac=21, Coef: 0.86, 95% CI: 0.44 to 1.29, $p=6.8x10^{-5}$) and three more missense variants. Variants associated with bilirubin were also predictors in untreated individuals (GMTE0 analysis in TWIST results).

Two variants were associated with increased ALT levels (12:21178996:G:A, mac=38 – Coef:0.48, 95% CI: 0.18 to 0.8, p= 0.002 and 12:21202664:G:A, mac=15 – Coef: 0.53, 95% CI: 0.04 to 1.03, p=0.03) in TWIST GMTE1 analysis, but not in GMTE0, suggesting a pharmacogenetic effect.

REFERENCES

1. Expert Panel on Detection, Evaluation, and Treatment of High Blood Cholesterol in Adults. Executive Summary of The Third Report of The National Cholesterol Education Program (NCEP) Expert Panel on Detection, Evaluation, And Treatment of High Blood Cholesterol In Adults (Adult Treatment Panel III). JAMA. 2001 May 16;285(19):2486–97.

2. Türkmen D et al. Statin treatment effectiveness and the *SLCO1B1* *5 reduced function genotype: Long-term outcomes in women and men. Brit J Clinical Pharma. 2022 Jul;88(7):3230–40.

3. Stewart A. SLCO1B1 Polymorphisms and Statin-Induced Myopathy. PLoS Curr. 2013 Dec 4;5:ecurrents.eogt.d21e7f0c58463571bb0d9d3a19b82203.
